# Strategic autonomy as a means to counter protectionism

**DOI:** 10.1007/s12027-021-00670-w

**Published:** 2021-06-23

**Authors:** Jens Hillebrand Pohl

**Affiliations:** Academy of European Law, Trier, Germany

The Covid-19 pandemic has touched every corner of Europe in a way not experienced for generations, certainly not in peacetime. With the virus having claimed hundreds of thousands of lives in Europe alone, it is difficult to imagine that things could actually have been even worse. Yet, the public health crisis unleashed by the pandemic can be seen as something of a warning shot; a test of our crisis readiness and our resilience.

The crisis has shaken us out of our complacency and made us realise how vulnerable our lives, livelihoods and lifestyles are to external disruption. The pandemic has also illustrated the necessity and risk of global interdependence, as well as the need for trusted partners and good neighbours. Put simply: to cope in an emergency, the nations of Europe have experienced the need to act as a team, put their differences aside, and assume collective responsibility for a most urgent priority. And with responsibility comes the need to be in control—to be autonomous, as a group of like-minded nations, in a strategic sense.

## Strategic autonomy: two words that can mean anything to anyone

The origin and historical use of the term ‘strategic autonomy’ is a poor guide to its current and evolving meaning in policy circles.^1^ Clearly something of a mantra these days, the term is best understood as what has been termed an ‘essentially contested concept’ in the philosophy of language;^2^ an attractive term that is frequently and enthusiastically used, but one without a common understanding of its precise meaning.

Nevertheless, the embrace of ‘strategic autonomy’ in the wider political discourse does signify a sincere recognition of certain key vulnerabilities that lie at the heart of the European project and a political will to do something about them. The work on the ‘Strategic Compass’ is certainly a very important and welcome development as it aims to embark on a process of forging a common methodology of threat analysis, which in turn is a starting point towards a ‘common language’ of security.^3^ That exercise will not define ultimate goals. It will not answer the crucial question ‘what do Europe’s strategic rivals want?’, nor indeed the equally crucial ‘what does Europe want?’. But it will facilitate an evolution towards a joint security process.

As such, the path ahead for the ‘strategic autonomy’ discourse is one of incrementalism, which is an approach that has ‘Europe’ written all over it. As many times in the past, incrementalism may pay off this time as well. But we also know—all too well—that Europe tends to be built through crises. And surely nothing short of a systemic jolt would be needed to turn strategic autonomy from project into reality.

## Protect *what*?

A common criticism of strategic autonomy as a political ambition is that it is little more than a thin guise for old-school protectionism.^4^ ‘Protection’ in this sense means protecting (politically well-connected) corporate managers of moribund industries from foreign competition and foreign takeovers.

Indeed, the mere term ‘protection’ strikes some as an ugly word in some contexts. Yet, reasonable folk will also agree that ‘protection’ as such is inherently neither good nor bad as an activity. It all depends on *what is being protected from what by whom for what purpose*.

Take Covid as an example. The pandemic crisis has provided a unique illustration of Europe’s vulnerabilities. Protecting the general public from a pandemic has emerged as a top priority across the continent. In this context, intervening in an emergency to protect for the purpose of securing the availability of critical capabilities and resources is clearly very different from protecting for the purpose of shielding underperforming corporations from foreign competitors.^5^

Being deprived of crisis capabilities implies the risk of becoming a political vassal—to Sputnik V’s Russia, Sinopharm’s China, or indeed the United States, to name a few. The localisation of first-rate pharmaceutical research and production in Europe is insufficient to guarantee the availability of critical medical supplies and resources, as the very public supply complications linked to the AstraZeneca covid-19 vaccine amply demonstrates.^6^ Effective investment screening and export control mechanisms are necessary in the face of vaccine nationalism and a breakdown of global coordination of vaccine deliveries. Well designed, such measures serve to uphold market mechanisms and provide clarity and predictability to vaccine producers, while de-politicising vaccine supply efforts.

Protecting critical capabilities presumes the existence of capabilities to protect, including critical technologies. Once lost, no amount of financial resources or political will could re-create a first-rate pharmaceutical industry in Europe on short notice. Fortunately, this industry has not been lost, but the same is not true in many other sectors and technologies, including digital technologies and most notably, artificial intelligence. Europe also lacks control over certain critical digital infrastructures. Imagine the sudden disappearance of services such as Amazon, Facebook and Google. Such loss may not, at first glance, seem critical, but would indeed prove highly disruptive to many people’s everyday lives. Such massive inconvenience is political leverage and matters in a democracy. Hence an argument could be made that such lack of critical digital infrastructure poses a risk to political security.

Projects and efforts such as GAIA-X and the battery alliance,^7^ the idea of an ‘Airbus for AI’,^8^ and the projected use of trade policy to promote onshoring of critical processes^9^ are not about cuddling up to fat-cat yoghurt bosses. A more valid criticism is that initiatives such as these should have already been conceived a decade ago.

At a deeper level, it is also important to be very clear about ‘good’ and ‘bad’ protection. The distinction lies in the ‘why’—the *telos*—the effective purpose of the protection. It turns out that the distinction is quite simple.

## Protecting against protectionism

An existential paradox of capitalism is that, on the one hand, businesses need the protection of society to exist, while, on the other hand, the very same protection may drive businesses out of existence. Without laws and courts to enforce contracts and to protect property rights that affect third parties, there can be no private enterprise. Public intervention is useful to protect against societal costs (negative externalities), such as pollution, or to protect societal benefits (positive externalities), such as research and development. Moreover, a healthy level of competition does not emerge in the absence of laws to protect competition and protect against unfair practices, whether originating internally or externally. Anarcho-capitalism is not associated with sound economic progress. Yet, over-regulation and over-protection can likewise be very damaging to competition and to the economic climate.

What counts as appropriate protection, as well as what counts as societal costs or benefits, are a reflection of society’s evolving values and interests. The British abolitionist movement in the early 1800s was motivated by a powerful sentiment of the inhumanity and moral repugnance of the slave trade and brought economic barriers to bear against the countries that persisted in this abomination, notably the pre-Civil War United States.^10^ Slave-produced American cotton constituted an unfair competitive practice, which made non-slave produced British cotton uncompetitive in world markets due to the higher price of the latter. Take also the more recent examples of child labour and prison labour. The banning of importation into the EU of products made by under-age children or political prisoners is not motivated by a desire to stimulate child labour or prison labour in Europe. Nor is banning environmentally hazardous products or blocking investments made by criminally obtained funds about European neo-mercantilism. Rather, it is about weeding out practices that undermine societal well-being. Enforcing the European Green Deal, notably the promotion of the EU taxonomy of climate neutral activities and the proposed carbon border adjustment tax, is thus not about giving a ‘hidden subsidy’ to European industry or about shielding Europe’s businesses from foreign competition, but about ensuring that European industry is not under-cut by unfair practices by unscrupulous producers beyond its jurisdiction.^11^

More nuanced—but no less serious—examples of unfair practices abound, particularly asymmetric practices that undermine the premise of free trade and free capital mobility. Because even the most ardent free traders admit that the promised benefit of free trade depends on some degree of reciprocity. If a trading partner consistently subsidises its export industry and discourages imports through red-tape, corrupt local practices, and lack of legal safeguards, trade will be lopsided.^12^ Any advantages of unilateral reduction of tariffs in the importing country and any advantages stemming from a legally predictable environment there will be captured by the exporting country. Free trade works only if everyone play by the same rules, with a shared understanding, not only on tariff levels, but much more importantly on so-called ‘non-tariff barriers’—everything ranging from banning the produce of slavery to technical safety standards, state aid, and competition rules. The Brexit negotiations illustrate this reality very vividly; without a level playing field there cannot be unimpeded trade.

Hence the pivotal twin realization: (1) There are certain (essentially non-economic) societal interests on which economic interests are predicated, linked to the notion of societal well-being, and (2) The economic benefits of openness rely on some level of reciprocity—which lies at the heart of the distinction between ‘good’ and ‘bad’ protection. In fact, protection against the latter kind of unfair practices (in other words, practices that undermine the benefits of economic openness) is the opposite to protectionism. It is protection against protectionism or *counter*-protectionism.

## Fighting fire with fire works

And yet, some might counter-argue by asking “is protecting against protectionism not like fighting fire with fire?”. The answer is yes, absolutely. Firefighters call it a ‘controlled burn’. It works.

The past 70 years of experience with the General Agreement on Tariffs and Trade demonstrates that free trade is a two-way street, and that reduction of trade barriers in pursuit of free trade did not occur spontaneously but required reciprocity and a strict process and regulation to materialize.^13^ By contrast, proponents of unilateral free trade cannot draw much comfort from history. The most notable example of free-trade unilateralism is the trade policy of Great Britain from the repeal of the Corn Laws and Navigation Laws in 1846 and 1849, respectively, until the 1920s.^14^ That policy made sense in the 1850s as a way to maintain a buyer’s market in raw materials and a seller’s market in manufactured goods. But when faced with a protectionist United States, a protectionist Imperial Germany and a protectionist Imperial Japan (all of which had nurtured their own domestic manufacturing capabilities), British manufacturing steadily—but slowly enough not to spark a reaction—lost out in global competition. Meanwhile British capital sought superior returns by investing in the (protected) new industries in these countries.

Unilateral free trade today does not just mean eliminating tariffs without reciprocity, but tolerating asymmetric terms of trade whereby a trading partner gains a competitive edge through unfair means that can range from unacceptable labour practices, climate degradation, uncontrolled epidemics, forced technology transfers, subsidies and so on. Unilateral free capital mobility means not only allowing the entry of capital from countries which themselves operate inward capital controls, but also not scrutinising the origin and destination of the inward and outward flow of capital.

It is noticeable in this context that, while we, today, are equipped with the tools to ensure reciprocity in trade, subject to international trade undertakings, including soon also an instrument to better tackle foreign subsidies, we are only belatedly weaponizing capital mobility. While have now have the first European framework for inward investment screening, we still lack a framework for screening outward investment. For example, we do not control whether European capital is used to finance hostile governments or economic practices involving human rights violations, environmental destruction, or other unacceptable commercial or industrial practices in third countries.

## Roadblocks on the journey towards European strategic autonomy

Assuming that the Strategic Compass can kick-start a constructive dialogue on what European strategic autonomy actually may and should mean, and assuming further that we can have a debate and lay to rest the misguided idea of unilateral free trade, what are the principal stumbling blocks on the road towards strategic autonomy? Four potential roadblocks stand out.

### Subject, object and purpose of international conflict

It is intuitive to think of international relations from a traditional and hierarchical view of States as actors on the international plane, distinct from its subjects that pursue their own separate non-state objectives. This view does not reflect geoeconomic reality, however. States are economic actors in their own right, something that is rarely contemplated in economics textbooks. Often states are run with faint insulation between state interest and the interests of those who govern them, which could be a royal family as in Saudi Arabia, an oil giant as in Gazprom’s Russia or a party organisation as in China. Understanding the intertwining of business interests, desired lifestyles of the elite and broader economic interests, as well as the role of the state as an instrument of furthering such interests is necessary to understand the motivations and agendas of Europe’s strategic environment. Linked to such a geoeconomic realism is the concept of the aims, scale and outcomes of conflict. Unlike ideologically inspired conflicts, there are other outcomes than *debellatio*. We should be interested in the type of economic settlements that may motivate potential future conflict.

### The future of international relations is domestic

Paraphrasing the title of Anne-Marie Slaughter’s and William Burke-White’s famous 2006 article, it is important that we do not get stuck on the road towards strategic autonomy in old-school thinking about foreign and defence policy.^15^

Too much can be made of the question of qualified-majority voting in CFSP (or indeed CSDP) matters. Perhaps change will come, at least in the areas of human rights, sanctions, missions and operations. But importantly, the EU already possesses all the essential tools it needs in the brave new world of economic statecraft, notably as concerns digital policy, high-tech industry and common commercial policy generally. CFSP including CSDP is only a part of the toolbox. Linked to this is the risk of a ‘conventional defence bias’ slowing down progress by not focusing attention on threats posed by non-state actors (such as third-country state-backed enterprises operating in Europe) and, much more importantly, by intra-state threats (ranging from the risk of outright corruption of European officials and judges to less obvious forms of influencing at the technocratic level and public-opinion operations). Such activities could definitely torpedo Europe’s goal of achieving strategic autonomy and should be clearly conceptualised and prioritised.

### Strategic autonomy does not mean ‘strategic autarky’

Those in the ‘anti-autonomy camp’, which might include the ‘New Hanseatic League’ countries, worry that strategic autonomy is a policy of self-sufficiency and isolationism, kept alive through massive subsidies. Without further clarification of what is meant by ‘strategic autonomy’, such worries are not completely unfounded.

Yet, if understood as anti-protectionism measures, it should be clear that Europe’s strategic autonomy is not only compatible with, but may be strengthened by, active cooperation with like-minded third-countries. The EU already has a formidable network of Free Trade Agreements (FTAs) with numerous countries around the world. Granted, many of these FTAs do not reflect sufficiently the evolved priority of combating unfair practices. Most post-Lisbon FTAs do have sustainable development chapters as well as special chapters on labour and environmental protections, but they surely need to be reviewed.

Nevertheless, it is readily imaginable that the ‘old normal’ of free trade and free capital mobility could very well be maintained vis-à-vis countries sharing a compatible understanding of today’s unfair practices, notably in the area of disease prevention and climate change, and about today’s geoeconomic challenges. It may even be possible to forge a broad-based transatlantic pact along those lines, particularly focusing on a common EU-U.S. understanding of technology threats, as well as with like-minded countries in the Indo-Pacific and elsewhere. One possibility that has been floated is to engage broadly with a coalition of leading democracies, such as the D-10 Group (G-7 plus the Republic of Korea, India and Australia).

### Post-trump synthesis

No future path for European strategic autonomy is imaginable without considering the impact of the Trump presidency, its legacy and continuing repercussions. It is clear that the Trump administration was not just an aberration—a freak accident of democracy. There is something real about the political force of over 70 million Americans voting for President Trump after four years of Trumpism in practice, which gives reason for pause.

The Biden presidency is not brushing these people off as ‘deplorables’ and ‘losers of globalization’, switching the autopilot back on and carrying on where the Obama Administration left off. Rather, it appears that safeguarding the economic security of the American nation is a paramount objective—both in relation to the economic security of the American people (especially the working class) and U.S. national economic security. A U.S. Green New Deal looks likely to become reality and may effectively result in a perpetuation of the process of decoupling of the U.S. economy from its dependence on Chinese imports.

Moreover, fixing the World Trade Organisation (WTO) will necessitate the unanimous agreement of all 164 members. One cannot reasonably expect a reinstatement of the old order any time soon. Europe is clearly not able to alone drive these developments, but it will be impacted, as will its journey towards strategic autonomy. As the United States is embarking on a new geoeconomic agenda, it would be far-fetched for Europe to unilaterally keep pursuing yesterday’s U.S. foreign economic policy agenda of unfettered free-trade multilateralism—which, by the way, Europe itself only gradually began to internalize in the 1990s—and forget about today’s more pressing issues of climate change, disease prevention, and both economic and cyber insecurity.

## Concluding reflections

Responding to the pandemic has been a rare example of a paramount objective shared across nations and across the political spectrum, even though there may be disagreement as to the best way of achieving that objective.

The intensity and intrusiveness of the pandemic experience has a pedagogical dimension that should not go to waste. It helps to activate our imagination. That imagination can be applied as a prism for other pressing issues of today, notably the climate crisis, allowing us to reflect on our vulnerabilities, visualise negative outcomes and to think about how to best prevent such outcomes.

What we should ask ourselves next is what are the structures or rigidities that will frame the ambitions for European strategic autonomy? Here, there is something to the critique (or fear) of the New Hanseatics: that the re-wiring of the European economy as a result of the grand initiatives associated with European strategic autonomy (such as the New Industrial Strategy, the European Green Deal, and the proposed Foreign Subsidies Regulation) will necessitate an economy powered by state aid and EU subsidization in various forms, including subsidised capital. However, Europe’s newfound determination to address investments into the EU that are subsidized by foreign powers may reveal that capital allocation is already a process fraught by distortions and that Europe’s strategic financial autonomy is a priority that needs to be elevated to the level of attention it deserves.

## Footnotes

1 On the origin of the term, see, e.g., Rafael García Pérez (2020) “Strategic Autonomy of the European Union: A Perspective”. In: E. Conde et al. (eds.) *The Routledge Handbook of European Security Law and Policy* (Routledge 2020). On it its current meaning, see, e.g., Nathalie Tocci (2021) “Towards European Cooperative Autonomy”. In: R. Haar, Th. Christensen, S. Lange and S. Vanhoonacker (eds.) *The Making of European Security Policy: Between Institutional Dynamics and Global Challenges* (Routledge 2021), and by the same author, (2021) *European Strategic Autonomy: What It Is, Why We Need It, How to Achieve It*. IAI – Instituto Affari Internazionali. Available (as of 10 May 2021) at: https://www.iai.it/sites/default/files/9788893681780.pdf; Daniel Fiott (2018) *Strategic Autonomy: Towards ‘European Sovereignty’ in Defence?* EU Institute for Security Studies. Available (as of 10 May 2021) at: https://www.iss.europa.eu/sites/default/files/EUISSFiles/Brief%2012__Strategic%20Autonomy.pdf.

2 Walter B. Gallie (1956) “Essentially Contested Concepts” LVI Proceedings of the Aristotelian Society: 167. For the development and application of the framework of essentially contested concepts to the notion of sovereignty, see Samantha Besson (2004) “Sovereignty in Conflict” European Integration 8(15): 7–16; Dan Sarooshi (2004) “The Essentially Contested Nature of the Concept of Sovereignty: Implications for the Exercise by International Organizations of Delegated Powers of Government” Michigan Journal of International Law 25: 1108–20.

3 Daniel Fiott (2020) *Unchartered Territory? Towards a Common Threat Analysis and a Strategic Compass for EU Security and Defence*. EU Institute for Security Studies. Available (as of 10 May 2021) at: https://www.iss.europa.eu/content/uncharted-territory-towards-common-threat-analysis-and-strategic-compass-eu-security-and.

4 See, e.g., “The Revenge of the Strategic Yoghurt”. *The Economist* (3 October 2020). Available (as of 10 May 2021) at: https://www.economist.com/europe/2020/10/03/the-revenge-of-strategic-yogurt.

5 See in this respect, European Commission (2020) Guidance to the Member States Concerning Foreign Direct Investment and Free Movement of Capital from Third Countries, and the Protection of Europe’s Strategic Assets, Ahead of the Application of Regulation (EU) 2019/452 (FDI Screening Regulation), Communication from the Commission, C(2020) 1981 final.

6 See Simon J. Evenett (2021) “Export Controls on COVID-19 Vaccines: Has the EU Opened Pandora’s Box?” Journal of World Trade 55(3): 397-408; Bianca Nalbandian (2020). “EU Foreign Direct Investment Screening in Pandemic Times: Between EU Protection and EU Protectionisms”. *blogdroiteuropeen*. Available (as of 10 May 2021) at: https://blogdroiteuropeen.com/2020/04/07/eu-foreign-direct-investment-screening-in-pandemic-times-between-eu-protection-and-eu-protectionisms-by-bianca-nalbandian/.

7 GAIA-X is a project for the development of an efficient and competitive, secure and trustworthy federation of data infrastructure and service providers for Europe, see (as of 10 May 2021): https://www.data-infrastructure.eu/GAIAX/Navigation/EN/Home/home.html; the European Battery Alliance is Europe’s plan to create its own competitive and sustainable battery cell manufacturing value chain, see (as of 10 May 2021): https://www.eba250.com.

8 “Germany seeks European cooperation on artificial intelligence”. *Reuters* (4 December 2018). Available (as of 10 May 2021): https://www.reuters.com/article/us-germany-tech-idUSKBN1O31EB.

9 Compare in this respect, European Commission, A New Industrial Strategy for Europe, 10 March 2020, COM(2020) 102 final, 2.

10 For an insightful study of the considerations that motivated British abolitionism, see David Brion Bavis (1999) *The Problem of Slavery in the Age of Revolution, 1770-1823* (Oxford University Press): 39-83.

11 This argument was elegantly made by Jagdish Bhagwati (1995) “Trade Liberalisation and ‘Fair Trade’ Demands: Addressing the Environmental and Labour Standards Issues,” The World Economy 18: 745–759.

12 See further Erdal Yalcin, Gabriel Felbermayr, and Luisa Kinzius (2017) *Hidden Protectionism: Non-Tariff Barriers and Implications for International Trade*. ifo Center for International Economics. ifo Institute (Leibniz Institute for Economic Research at the University of Munich). Available (as of 10 May 2021) at: https://www.cesifo.org/DocDL/ifo_Forschungsberichte_91_2017_Yalcin_etal_Protectionism.pdf.

13 For a comprehensive analysis on the history of the GATT and the ITO, see Robert E. Hudec (1990) *The GATT Legal System and World Trade Diplomacy* (2nd ed., Cambridge University Press).

14 See, e.g., Marc-William Palen (2010). “Protection, Federation and Union: The Global Impact of the McKinley Tariff upon the British Empire, 1890-94”. Journal of Imperial and Commonwealth History. 38 (3): 395–418; cf. Paul Bairoch (1989) “European Trade Policy, 1815–1914”. In: P. Mathias and S. Pollard (eds.) *The Cambridge Economic History*, *vol. VIII: The Industrial Economies: The development of Economic and Social Policies* (Cambridge University Press): 69.

15 Anne-Marie Slaughter and William Burke-White (2006) “The Future of International Law Is Domestic (or, The European Way of Law)”. Harvard International Law Journal 47(2): 327-352.

